# Influence of Specimen Preparation and Test Methods on the Flexural Strength Results of Monolithic Zirconia Materials

**DOI:** 10.3390/ma9030180

**Published:** 2016-03-09

**Authors:** Christine Schatz, Monika Strickstrock, Malgorzata Roos, Daniel Edelhoff, Marlis Eichberger, Isabella-Maria Zylla, Bogna Stawarczyk

**Affiliations:** 1 Department of Prosthodontics, Dental School, Ludwig-Maximilians-University Munich, Goethestrasse 70, Munich 80336, Germany; c.sa.schatz@googlemail.com (C.S.); daniel.edelhoff@med.uni-muenchen.de (D.E.); marlis.eichberger@med.uni-muenchen.de (M.E.); 2 Faculty of Engineering and Computer Science, Material Science and Analysis, University of Applied Sciences, Hochschule Osnabrück, Albrechtstrasse 30, Osnabrück 49076, Germany; monika.strickstrock@hs-osnabrueck.de (M.S.); i.m.zylla@hs-osnabrueck.de (I.-M.Z.); 3 Department of Biostatistics, Epidemiology Biostatistics and Prevention Institute, University of Zurich, Hirschengraben 84, Zurich 8001, Switzerland; malgorzata.roos@uzh.ch

**Keywords:** monolithic zirconia, flexural strength, biaxial strength, 3-point flexural strength, 4-point flexural strength, specimen preparation

## Abstract

The aim of this work was to evaluate the influence of specimen preparation and test method on the flexural strength results of monolithic zirconia. Different monolithic zirconia materials (Ceramill Zolid (Amann Girrbach, Koblach, Austria), Zenostar ZrTranslucent (Wieland Dental, Pforzheim, Germany), and DD Bio zx^2^ (Dental Direkt, Spenge, Germany)) were tested with three different methods: 3-point, 4-point, and biaxial flexural strength. Additionally, different specimen preparation methods were applied: either dry polishing before sintering or wet polishing after sintering. Each subgroup included 40 specimens. The surface roughness was assessed using scanning electron microscopy (SEM) and a profilometer whereas monoclinic phase transformation was investigated with X-ray diffraction. The data were analyzed using a three-way Analysis of Variance (ANOVA) with respect to the three factors: zirconia, specimen preparation, and test method. One-way ANOVA was conducted for the test method and zirconia factors within the combination of two other factors. A 2-parameter Weibull distribution assumption was applied to analyze the reliability under different testing conditions. In general, values measured using the 4-point test method presented the lowest flexural strength values. The flexural strength findings can be grouped in the following order: 4-point < 3-point < biaxial. Specimens prepared after sintering showed significantly higher flexural strength values than prepared before sintering. The Weibull moduli ranged from 5.1 to 16.5. Specimens polished before sintering showed higher surface roughness values than specimens polished after sintering. In contrast, no strong impact of the polishing procedures on the monoclinic surface layer was observed. No impact of zirconia material on flexural strength was found. The test method and the preparation method significantly influenced the flexural strength values.

## 1. Introduction

Y-TZP (Yttria partially stabilized tetragonal zirconia) Zirconia has become of interest in dentistry, because of its high flexural strength [1] and its well-known transformation toughening ability [2]. Conventional zirconia (3Y-TZP: Yttrium-cation-doped tetragonal zirconia polycrystals, typically 2–3 mol% Y_2_O_3_) shows translucency on human dentin level and is therefore not suitable for monolithic tooth restorations from the esthetic point of view [3].

Modifications of the microstructure and composition were conducted in recent years to accomplish an adequate esthetic appearance for full anatomical zirconia restorations. For example, the Al_2_O_3_ percentage was reduced from 0.25 to 0.05 wt % and customized with a smaller grain size. In addition, the Al_2_O_3_ grains were positioned on the grain boundaries of the ZrO_2_ grains. The survival time of all-ceramic systems is strongly related to the flexural strengths of the restoration, which is the result of the flexural strength of core and veneering material, and the bond strength between both materials [4,5]. Although excellent strengths were reported for the zirconia core material, many studies indicate fractures in the veneering ceramic [6,7]. To avoid this problem and to facilitate the fabrication process, the demand for zirconia restorations in a monolithic design without veneering has increased [8]. Different studies reported on a higher fracture resistance of monolithic zirconia crowns compared to conventional veneered versions [8,9].

Many test methods have been introduced to measure the flexural strength of brittle ceramic materials [10,11]. Among them biaxial, 3-point testing and 4-point testing have evolved to be the most common. In 3-point testing a non-uniform stress field under the loading piston is created, whereas in 4-point testing the stress field between the support rolls is uniform, which can lead to different flexural strength findings. Variations in specimen sizes and testing-methods lead to different flaw size populations and thus different strength measurements [11,12]. The problem with brittle ceramic materials is that inherent material inhomogeneity can induce flaws such as micro-cracks or grain pullouts throughout the volume or on the surface of a material. This can lead to catastrophic failure. The Weibull distributional assumption with its parameters Weibull modulus m and characteristic strength s tries to take the largest flaw population into account. Whereas s corresponds to the 63.2% failure probability, a high Weibull modulus m is associated with a higher reliability of the material.

Regarding surface preparation methods, several studies showed that the preparation of the specimen has an important influence on the obtained flexural strength data [13,14,15,16,17,18]. Therefore, the aim of this study was to investigate the influence of specimen preparation and the test method on the flexural strength and Weibull statistics of different monolithic zirconia materials. The underlying null hypothesis was that zirconia materials, the specimen preparation methods, as well as the flexural strength test methods do not influence the flexural strength values and the reliability of monolithic zirconia materials.

## 2. Material and Methods

Three different pre-sintered zirconia materials for monolithic restorations were tested in this study: Ceramill Zolid (C), Zenostar Zr (Z), and DD Bio zx^2^ (D) (Table 1).

Two different polishing methods were applied. Manually dry polishing before sintering and machine wet polishing after sintering. The zirconia groups (C, Z, D/dry and wet polished) were tested for biaxial, 3-point or 4-point flexural strength. Each group contained 40 specimens. In summary, 720 zirconia specimens were fabricated (Table 2).

### 2.1. Specimen Preparation

All specimens were prepared in partially sintered state and in an enlarged size to compensate sintering shrinkage. The specimen for biaxial flexural strength measurement required a disc shape with a diameter of 16 mm. A CAM (computer aided manufacturing) machine (I-Mes 4030, Wieland Dental + Technik, Pforzheim, Germany) was used to mill cylinders out of the zirconia blanks (Software: Zenotec CAM 3.2 advanced V2.2017, Wieland Dental + Technik). A low speed (approximate 2.5 m/s) diamond saw (Well Diamantdrahtsägen, Mannheim, Germany; thread: type A 3–3) was used to cut the cylinders into slices. The saw uses a fine 0.3 mm diamond thread with embedded diamonds (diameter approximate 60 µm), the applied pressure was approximately 50 g. The specimens, for 3-point and 4-point flexural strength measurement were cut directly from the zirconia blanks with the diamond saw.

Manual polishing before sintering was done with SiC discs (Struers, Ballerup, Denmark). The sequence was SiC P400, SiC P500, and SiC P1000. The grinding time was 5 s per specimen-side and SiC disc. The load was applied by finger pressure and manual polishing was performed in small circles.

Machine polishing after sintering was performed with a water-cooled polishing machine (Struers Abramin, Struers, Ballerup, Denmark). Up to six specimens were polished during a polishing cycle at the same time. The polishing protocol consisted of coarse grinding with diamond pads of 40 µm and 20 µm for 6 min per each side (Pads: Code Granu 40 µm, Code Graku 20 µm, Struers; speed: 150 rpm; applied pressure: 20 N) and fine polishing with subsequent polishing solutions and a polishing plate for 6 min per side (Plate: MD-Largo (Struers, Ballerup, Denmark); Solutions (which are purely water-based diamond suspensions): Dia Pro Allegro/Largo (9 µm) and Dia Pro Largo (3 µm), Struers, Ballerup, Denmark). High polishing was conducted for 30 s per side, again with a polishing plate and polishing solution (Plate: MD-Chem (Struers, Ballerup, Denmark); Solution (colloidal silica suspension for final polishing): OP-S, Struers, Ballerup, Denmark; speed 150 rpm; applied pressure 200 N).

The zirconia materials were sintered (density from 3 g/cm^3^ presintered to 6 g/cm^3^ fully sintered) in a universal sintering oven (Nabertherm, Lilienthal/Bremen, Germany), according to each manufacturer’s instructions (Table 3). The final dimensions DIN EN ISO 6872:2008 [20] were: for biaxial flexural strength measurement 16 mm × 1.2 mm (±0.05 mm), for 3-point flexural strength measurement 1.2 mm × 4 mm × 25 mm (±0.05 mm) and for 4-point flexural strength measurement 3 mm × 4 mm × 45 mm (±0.05 mm).

### 2.2. Flexural Strength Testing

**Figure 1:**
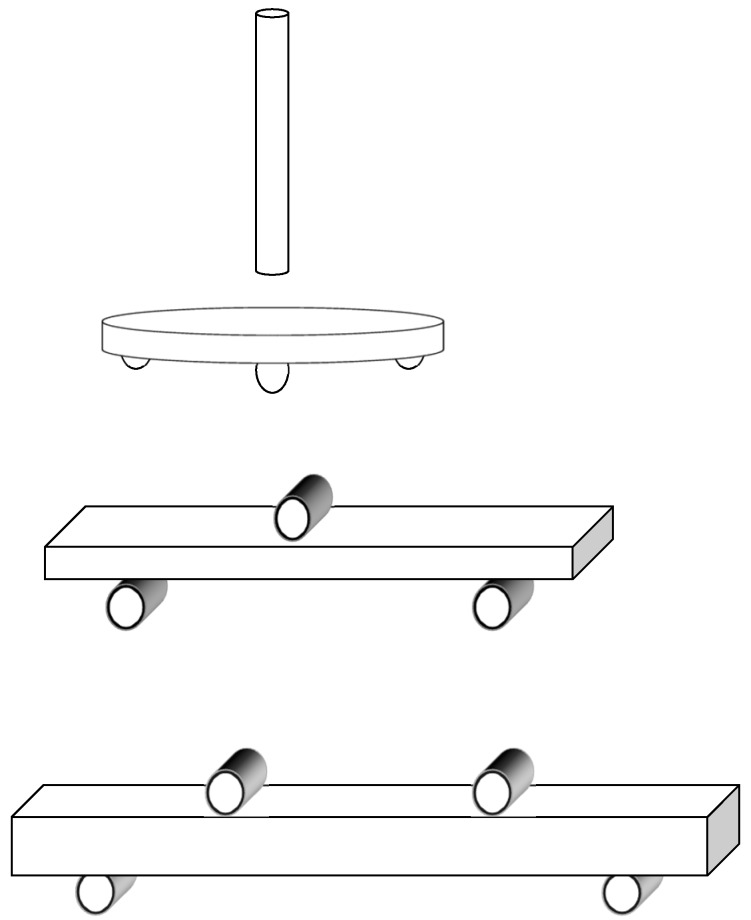
Schematic set up of biaxial; 3-point; 4-point test method.

Biaxial, 3-point, and 4-point flexural strength testing (Figure 1) are based upon DIN EN ISO 6872:2008 [20]. An appropriate sample holder was used for each specimen group to place it in a Universal Testing Machine (Zwick, Ulm, Germany) at a crosshead speed of 1 mm/min until failure. The specimens were tested dry at room temperature and dimensions were measured with a digital micrometer (Mitutoyo Deutschland, Neuss, Germany) to a precision of 0.01 mm.

The sample holder for the biaxial flexural strength test comprised three tempered steel balls with a diameter of 3.2 mm. The steel balls formed an equilateral triangle with an edge length of 10 mm and the ball support circle was 120°. The center of the specimens, which were put upon the steel balls and the center of the equilateral triangle were aligned coaxially. After the positioning the specimen’s center was loaded from above with a plunger with a diameter of 1.4 mm until failure. The flexural strength was calculated according to the formula σ = −0.25 N(X−Y)/d^2^ (σ: flexural strength; N: fracture load; coefficients X and Y with:

X = (1 + υ)ln[(r2⁄r3)]^2^ + [(1 − υ)⁄2](r2⁄r3)^2^

Y = (1 + υ)[1 + ln(r1⁄r3)^2^] + (1 − υ)(r1⁄r3)^2^

where υ: Poisson’s ratio (=0.25); r1: support (mean) contact diameter (mm); r2: (mean) loaded contact diameter (mm); r3: diameter of the specimen (mm); and d: thickness of the specimen (mm).

For the 3-point flexural strength measurement the specimen was placed on two tempered steel support rolls (diameter 1.6 mm). The distance between the two support rolls was 15 mm. Now a plunger (diameter 1.6 mm) loaded the specimen until failure. The formula σ = 3Nl/(2bd^2^) (σ: flexural strength; N: fracture load; l: distance between supports (mm); b: width of the specimen (mm); d: thickness of the specimen (mm)) was used to calculate 3-point flexural strength measurement.

The specimen for the 4-point flexural strength testing was placed on two tempered steel support rolls (diameter 4.0 mm) with a distance of 40 mm between the exterior supports. The plunger apparatus, consisting of two tempered steel rolls with a distance of 20 mm applied force until the specimens’ failure. The calculation formula was σ = 3Nl/(4bd^2^) (σ: flexural strength; N: fracture load; l: mid to mid distance between exterior supports (mm); b: width of the specimen (mm); d: thickness of the specimen (mm)).

### 2.3. Profilometry

A profilometer (MarSurf 400 SD26, Mahr, Göttingen, Germany) was used to detect surface roughness (R_a_) after preparation procedure. Six measurements for two specimens for each zirconia, test and polishing method were recorded (λ_c_ = 0.25 mm; L_t_ = 1.750 mm; pressure 0.7 mN) and a mean value was computed.

### 2.4. Scanning Electron Microscopy (SEM)

Scanning electron microscopy (EVO MA 10, Zeiss, Oberkochen, Germany) was used to assess the influence of specimen preparation on surface topography (15 kV, 150 mA, working distance of 10–12 mm). The specimens (*n* = 3 per group) were ultrasonically cleaned (Sonorex RK 100H; Bandelin, Berlin, Germany) and gold coated (Sputter Coater SC7640, Quorumtech, Newhaven, UK) before SEM examination.

### 2.5. X-ray Diffraction (XRD)

X-ray diffraction analysis (D8 Advance, Bruker AXS, Karlsruhe, Germany; settings: Bragg-Brentano Geometrie, Lynxeye-Dector in 1D-mode, nickel-kβ-filter) was employed to identify surface phase transformations. For each zirconia material, polishing and test method three specimens underwent XRD. Diffraction data were collected with Cu-Kα rays from the 2θ range between 25° and 40° with 40 kV, 40 mA and a step size of 0.02° and a scan time of 1.0 s/step, specimen rotation was 60 rpm. Under this condition of measurement the analyzed layer was 4.0–6.5 μm. The relative amount of monoclinic phase was calculated by means of the Garvie-Nicholson Equation modified through Toraya [21] (Equation (1)) using the maximum intensities of the reflexes I_t_(101), I_m_$\left(\bar{1}11\right),$ und I_m_(111).

(1) $$X_{m}=\frac{I_{m}\left(\bar{1}11\right)+I_{m}\left(111\right)}{I_{m}\left(\bar{1}11\right)+I_{m}\left(111\right)+I_{t}\left(101\right)}$$

The volume fraction (V_m_) is:

(2) $$V_{m}=\frac{1.311\times X_{m}}{1+0.311\times X_{m}}$$

### 2.6. Statistical Analysis and Methods

The power analysis was calculated in R (R, R Development Core Team, The R Foundation for Statistical Computing) using the data from our previous studies [22,23]. A sample size of 40 in each group will have 99% power to detect the difference of 200 MPa between two different flexural strength methods assuming that the common standard deviation is 178 MPa using the Students two sample t-test with the 0.017 two-sided significance level obtained after the Bonferroni correction (three tests between three different flexural strength methods within one zirconia material).

Anderson-Darling goodness-of-fit estimates were computed and the best fitting distributional assumption (normal or Weibull) was indicated by its smaller value. In addition, the assumption of normality of the data was investigated by means of the Kolmogorov-Smirnov test. Descriptive statistics mean, standard deviation (SD), and coefficient of variation (COV = SD/mean) together with the 95% confidence intervals (95% CI) for the mean were computed. A three-way ANOVA for flexural strength with respect to three factors: test method (TM: biaxial, 3-point, 4-point), specimen preparation (SP: before, after sintering), and zirconia material (ZM: C, Z, D) was computed using SPSS Version 22.0 (IBM Deutschland GmbH, Ehningen, Germany). These results were amended by one-way ANOVAs for test method and zirconia material factors within the combination of two other factors, separately. The two-sample t-test with Welch adjustment for differing variances was used to identify the influence of treatment within the combination of two other factors. In addition, the two-parameter Weibull distributional assumption was used to compute Weibull parameters (modulus = m and characteristic strength = s) with Least Squares and median rank plotting positions assumption [24,25]. Equal m, s, SD, and mean Bartlett’s modified likelihood ratio tests together with the appropriate Bonferroni post-hoc confidence interval were conducted with Minitab Version 14 (Minitab Ltd., Coventry, UK). Associations between two continuous variables were characterized by the non-parametric Spearman’s rho correlation. Results of statistical analysis with p-value smaller than 0.05, were considered to be statistically significant.

## 3. Results

Table 4 depicts the Anderson-Darling goodness-of-fit estimates.

The Kolmogorov-Smirnov test showed that not all of the data were normally distributed (Table 5).

**Figure 2:**
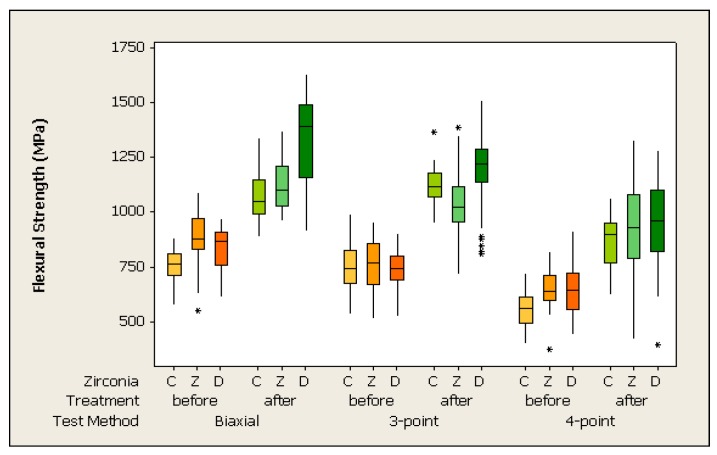
Influence of specimen preparation test method and zirconia material using different flexural strength testing methods.

Additionally, the 2-parameter Weibull analysis was used to describe the flexural strength (Table 6). The data are presented in Figure 2. The 3-way ANOVA observed the impact of specimen preparation method on the Weibull modulus: Zirconia C specimen tested in 3-point flexural strength had a significant higher Weibull modulus when polished after sintering (*p* < 0.001).

### 3.1. Influence of Test Method

The 4-point flexural strength test method indicated the lowest flexural strength values in all groups, regardless of which zirconia material was tested and which specimen preparation method was used (*p* < 0.001). An exception showed Z zirconia material polished after sintering. In this group 4-point and 3-point results were in the same range of values, but significantly lower than the biaxial values.

The biaxial strength test method provided the highest strength values. However, it was not significantly different from the 3-point test within the wet polished groups and within the dry polished zirconia C specimen (*p* > 0.05).

Regarding the Weibull modulus, 3-point testing showed a significantly higher Weibull modulus for the wet polished zirconia C group than for 4-point testing and biaxial testing (*p* < 0.001). Specimens tested in biaxial flexural strength presented a significantly higher Weibull modulus for wet polished Zirconia Z than those tested in 4-point strength test methods (*p* < 0.001). Other groups showed no influence of test method on the Weibull modulus (*p* > 0.05).

### 3.2. Influence of Specimen Preparation

The different polishing procedures greatly influenced the measured mean flexural strength independently of which zirconia was tested and which test method was applied. After sintering the wet polished specimen produced significantly higher flexural strength than specimens polished before sintering (*p* < 0.001). Only one group showed the impact of the specimen preparation method on the Weibull modulus. Zirconia C specimens tested in 3-point flexural strength (*p* < 0.001) provided a higher Weibull modulus when polished after sintering.

### 3.3. Influence of Tested Zirconia Materials

No impact of zirconia material on flexural strength values was found for 3-point tested dry polished specimen and 4-point tested wet polished specimen (*p* = 0.671).

The biaxial test method showed considerably lower values for dry polished zirconia C material than for material Z and D (*p* < 0.001). In contrast, wet polished Zirconia D presented noticeably higher flexural strength values compared to C and Z (*p* = 0.034).

Within specimens polished after sintering and tested in 3-point flexural strength, material Z showed significantly lower values than C and D (*p* = 0.046). Within specimens polished before sintering and tested in 4-point flexural strength, material C indicated significantly lower values than Z (*p* < 0.001). D was not different to Z. It is difficult to discern patterns in the ZM effect, as they seem to act differently for each TM and SP combination.

An impact of tested zirconia materials on Weibull modulus was observed only for specimens prepared after sintering and tested in 3-point test. In this group, a significantly higher Weibull modulus for C than for Z and D was observed (*p* < 0.001). All other groups were in the same Weibull modulus range.

### 3.4. Surface Roughness (Profilometer) and Surface Topography (SEM)

**Figure 3:**
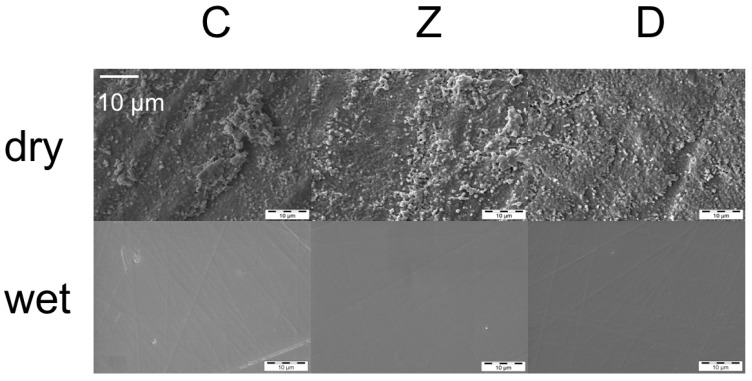
Scanning electron microscopy (SEM) pictures of dry and wet ground zirconia materials.

Dry polished specimens showed higher surface roughness compared to wet polished specimens. The surface roughness *R*_a_ values for the dry polishing method range from 0.31 to 0.41 μm and for the wet polishing method from 0.011 to 0.014 μm. An impact of zirconia materials (C, Z, D) was not observed. SEM pictures confirmed the results of the profilometric measurement (Figure 3). Roughness of the tested specimen was found to be associated with Weibull modulus (m) (Spearman’s rho correlation = 0.199, *p* = 0.428).

### 3.5. Characterization of Monoclinic Phase Transformation (X-ray Diffraction)

**Figure 4:**
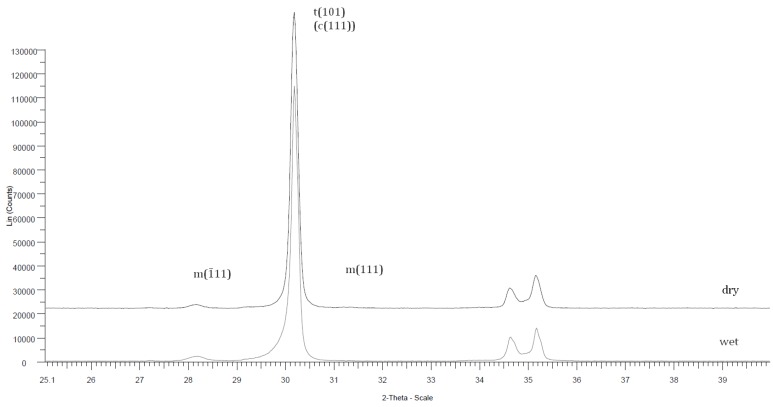
X-ray diffraction pattern example: Z, biaxial dry and wet.

The volume fraction of the monoclinic phase at the surface was low (∼2%) for all tested specimens (Figure 4), no material was noticeable different.

## 4. Discussion

To the knowledge of the authors, no data for monolithic zirconia are existent comparing the different flexural strength test methods. For the discussion of our data we will also consider studies with other ceramic materials.

The tested null hypothesis was that the flexural strength test methods would show no impact on strength values. This has to be rejected. It could be clearly discriminated between biaxial and 4-point tested groups, which presented the lowest flexural strength findings. In 4-point flexural strength testing, a larger area of the material is involved in stress application. Thus, the probability of crack initiation, reflected by lower flexural strength, is higher than for the 3-point test method [10]. In our data, 4-point tested groups were in all but one group statistically different from 3-point tested groups. This is in accordance with the data of a previous study, where veneering ceramics for zirconia have been tested [26].

In another study with zirconia core materials, biaxial strength testing resulted in higher and statistically different values compared to those, measured by 3-point flexural strength testing [27]. Except for one group, our data showed the highest mean biaxial flexural strength values, but we could only observe a clear discrimination between 3-point and biaxial testing in two groups. The high values in the biaxial test can be related to the negligible effect of undesirable edge failures [28,29]. Jin *et al.* [30] used all of the three test methods on different ceramic types. Although no zirconia material was included in this study, the authors also identified the lowest values for 4-point flexural strength measurement and could not clearly differentiate between biaxial and 3-point testing. A ranking order of the different flexural strength test methods can be described as the following: biaxial > 3-point > 4-point flexural strength measurement. Regarding the influence of the test method on the Weibull modulus it was observed that only wet polished, 3-point tested zirconia C and wet polished, 4-point tested zirconia Z had a higher Weibull modulus compared to other groups. Neither surface roughness nor SEM pictures can explain these findings.

The second tested hypothesis was, that the specimen preparation methods do not influence the flexural strength and the reliability of monolithic zirconia. The inherent reliability of the material can be described with the Weibull modulus. A high Weibull modulus is associated with a statistically higher reliability. It was found that specimen preparation after sintering showed significantly higher flexural strength values whereas no general trend for the Weibull moduli was observed. The Weibull modulus for 3-point tested Zirconia C polished after sintering was even higher, than for those polished before sintering. Thus the first part of the second hypothesis could not be confirmed. Regarding the reliability, the other part of the hypothesis is accepted within flexural strength test-groups and zirconia material. In contrast to that, a statistically lower Weibull modulus was noticed in wet polished 4-point tested groups for Zirconia C and Z. This indicates that polishing may induce flaws, but the probability of critical flaw detection increases, when a larger volume of the material is involved in the testing method. As failure is initiated by the largest flaw or element, this effect is also referred to as the weakest link hypothesis [31]. The authors believe that surface scratches shown in the SEM pictures, functioned as crack origin and led to significantly lower strength values of the 4-point tested specimen. The values for Weibull moduli ranged between (5.1 and 16.5), which is in accordance with the values reported for Y-TZP core materials tested in other studies [25,32]. In the present literature, contradictory findings are reported for flexural strength of zirconia materials, dependent on different surface alteration methods after the sintering process. Several authors described a decrease in flexural strength and Weibull modulus of zirconia, when the surface underwent a grinding process [14,18,33,34]. On the other hand, an increase in mean flexural strength and a decreased reliability was observed with the use of fine-grained diamond burs [35] and with wet hand grinding [36] although in this study the Weibull distribution was not discussed. When corund-blasting as a surface method is conducted, an increase of strength is also reported associated with a reduction of the Weibull modulus [18]. The increase in flexural strength can be explained with the phase transformation in zirconia materials. The amount of the monoclinic phase was found to rise, when the surface was treated after sintering [18,37]. This well-known transformation toughening mechanism of zirconia materials can lead to compressive surface stresses and thus elevate the flexural strength [38,39]. In contrast to that, in the present study no higher fraction of monoclinic zirconia on the surface of wet polished specimen was found. Thus the polishing procedure must have removed the layer of monoclinic zirconia. This effect was also observed by Guazatto *et al.* [17]. The above discussion of possible origins of the flaws is supported by Anderson-Darling (AD) goodness of fit estimates in Table 4. Smaller AD values indicate a better fit of the data by the assumed distribution. The authors were unable to find a single distributional assumption of flexural strength values fitting optimally for all test configurations. However, both Weibull and normal assumptions provided reasonable and useful approximations to compare all test groups. This conclusion is supported by a strong negative association between Weibull moduli (m) and COV (rho = −0.972, *p* < 0.001). Weibull modulus estimates ranged from 5.1 to 16.5 whereas those of COV from 0.077 to 0.223 indicating that both Weibull and normal assumptions were applicable to all test groups.

The third hypothesis was that different zirconia materials do not influence flexural strength. This has to be accepted, as a general trend as the zirconia materials could not be identified. It was observed that the zirconia materials used in this study act differently dependent on flexural strength test and treatment methods. Literature shows [19] that D contains a significantly larger grain size compared to zirconia C and Z. However, no association between grain size and all tested parameters was found in our study.

## 5. Conclusions

Within the limitations of the laboratory investigation, the following conclusions can be drawn:

(1) The 4-point flexural strength testing shows the lowest flexural strength data; biaxial test method the highest.

(2) The specimen preparation method significantly impacts the flexural strength findings; surface roughness was higher with dry polished specimens.

(3) Flexural strength values of tested zirconia materials range within the same values and no clear effect of the zirconia material could be observed.

## Figures and Tables

**Table 1:** Summary of materials used in the present study, their manufacturer with LOT number, chemical components, and grain size.

Abbreviations	Zirconia Materials	Manufacturers	LOT Number	Chemical Components (%)	Grain Size (μm^2^) Mean ± SD [19]
C	Ceramill Zolid	Amann Girrbach, Koblach, Austria	1111813	ZrO_2_ + HfO_2_ + Y_2_O_3_ > 99; Y_2_O_3_: 4.5–5.6; HfO_2_ < 5; Al_2_O_3_ < 0.5	0.088 ± 0.004 ^a^
Z	Zenostar Zr Translucent	Wieland+Dental, Pforzheim, Germany	20120306-27	ZrO_2_ + HfO_2_ + Y_2_O_3_ > 99; 4,5 < Y_2_O_3_ ≤ 6; HfO_2_ ≤ 5; Al_2_O_3_ + other oxides ≤ 1	0.092 ± 0.003 ^a^
D	DD Bio zx^2^	Dental Direkt, Spenge, Germany	30712803	ZrO_2_ + HfO_2_ + Y_2_O_3_ > 99; Al_2_O_3_ < 0.5; other oxides ≤ 1	0.124 ± 0.006 ^b^

^a,b^ Different letters present significant differences between materials.

**Table 2:** Division of specimens with abbreviation used. TM: test method, SP: specimen preparation, ZM: zirconia material.

Total	ZM	TM	SP	Subgroup
*N* = 720	C, D, Z *n* = 240	biaxial	dry-polished before sintering	*n* = 40
		*n* = 80	wet-polished after sintering	*n* = 40
		3-point	dry-polished before sintering	*n* = 40
		*n* = 80	wet-polished after sintering	*n* = 40
		4-point	dry-polished before sintering	*n* = 40
		*n* = 80	wet-polished after sintering	*n* = 40

**Table 3:** Sintering parameters of all tested zirconia materials used in this study.

ZM	Heat Rate (°C/h)	Holding Temperature and Time (°C, h)	Final Temperature (°C)	Holding Time (h)	Cooling Rate (°C/h)
C	480	-	1450	2	300
Z	600	900; 0.5 h; further with 200 °C/h	1450	2	600
D	480	900; 0.5 h; further with 200 °C/h	1450	2	600

**Table 4:** Anderson-Darling goodness of fit estimates.

TM	SP	ZM	Weibull	Normal	Optimal Fit	Optimal Fit Distribution
**Biaxial**	before	C	0.637	0.702	0.611	3-par Weibull
		Z	0.793	0.821	0.726	Logistic
		D	1.102	1.735	0.947	SEV
	after	C	2.405	0.799	0.482	3-par Log normal
		Z	2.070	0.713	0.593	3-par Log normal
		D	1.111	1.589	0.940	SEV
**3-point**	before	C	0.916	0.455	0.434	3-par Log normal
		Z	0.613	0.594	0.536	3-par Weibull
		D	0.577	0.508	0.480	3-par Weibull
	after	C	1.101	0.568	0.512	Logistic
		Z	2.053	0.942	0.669	3-par Log logistic
		D	0.946	1.412	0.835	3-par Weibull
**4-point**	before	C	1.024	0.488	0.472	3-par Log normal
		Z	0.946	0.746	0.743	3-par Log normal
		D	0.755	0.496	0.496	normal
	after	C	0.693	1.033	0.691	SEV
		Z	0.685	0.708	0.708	normal
		D	0.625	0.698	0.625	Weibull

**Table 5:** Descriptive statistics for flexural strength values of all measured groups. TM: test method; SP: specimen preparation; ZM: zirconia material; SD: standard deviation; 95% CI: 95% confidence interval; Min: minimum; Max: maximum; COV: coefficient of variation (%). If not otherwise indicated all values are presented in MPa.

TM	SP	ZM	SD	95% CI (SD)	Mean	95% CI (Mean)	COV %
**Biaxial**	before	C ^a/A^	81 ^a^	(64;102) ^a^	757 ^A^	(731;782) ^B^	11
		Z ^a/B^	115 ^a^	(91;144) ^a^	891 ^A^	(855;927) ^C^	13
		D ^a/B^	101 ^a^	(80;126) ^a^	835 *^,A^	(803;866) ^C^	12
	after	C ^a/A^	115 ^a^	(91;145) ^a^	1077 ^B^	(1040;1112) ^B^	11
		Z ^ab/A^	116 ^a^	(92;146) ^a^	1126 ^B^	(1090;1162) ^B^	10
		D ^b/B^	212 ^b^	(170;265) ^a^	1322 *^,B^	(1256;1388) ^B^	16
**3-point**	before	C ^a/A^	110 ^a^	(87;139) ^a^	752 ^A^	(718;787) ^B^	15
		Z ^a/A^	124 ^a^	(98;156) ^a^	755 ^A^	(716;793) ^B^	16
		D ^a/A^	97 ^a^	(76;122) ^a^	743 ^A^	(712;773) ^B^	13
	after	C ^a/AB^	86 ^a^	(68;108) ^a^	1118 ^B^	(1090;1144) ^B^	8
		Z ^ab/A^	156 ^a^	(123;196) ^a^	1039 ^B^	(990;1087) ^AB^	15
		D ^b/B^	173 ^a^	(138;216) ^a^	1183 *^,B^	(1129;1237) ^B^	15
**4-point**	before	C ^a/A^	78 ^a^	(61;99) ^a^	561 *^,A^	(536;585) ^A^	14
		Z ^a/B^	88 ^a^	(69;110) ^a^	646 ^A^	(618;673) ^A^	14
		D ^a/AB^	112 ^a^	(88;141)^a^	637 ^A^	(602;672) ^A^	18
	after	C ^a/A^	122 ^a^	(97;153)^a^	873 ^B^	(834;911) ^A^	14
		Z ^a/A^	206 ^b^	(163;260) ^a^	922 ^B^	(858;987) ^A^	22
		D ^a/A^	182 ^a^	(144;228) ^a^	947 ^B^	(890;1004) ^A^	19

* not normally distributed; ^abc^ significant differences for SD values; column ZM indicates significant difference between ZM within TM and SP; column SD indicates significant difference between SP within TM and ZM; column 95% CI (SD) indicates significant difference between TM within SP and ZM; ^ABC^ significant differences for mean values; column ZM indicates significant difference between ZM within TM and SP; column mean indicates significant difference between SP within TM and ZM; column 95% CI (mean) indicates significant difference between TM within SP and ZM.

**Table 6:** Weibull statistics for flexural strength values of all measured groups. TM: test method; SP: specimen preparation; ZM: zirconia material; m: Weibull modulus; 95% CI: 95% confidence interval; s: characteristical strength. All values are presented in MPa.

TM	SP	ZM	m	95%CI (m)	s	95%CI (s)
**Biaxial**	before	C ^a/A^	11.2 ^a^	(8.7;14.4) ^a^	791 ^A^	(768;814) ^A^
		Z ^a/B^	9.0 ^a^	(6.9;11.6) ^a^	940 ^A^	(906;975) ^C^
		D ^a/B^	9.6 ^a^	(7.1;13.1) ^a^	878 ^A^	(848;908) ^C^
	after	C ^a/A^	12.4 ^a^	(10.5;14.6) ^a,b^	1120 ^B^	(1090;1152) ^B^
		Z ^a/A^	12.8 ^a^	(10.7;15.2) ^b^	1170 ^B^	(1139;1202) ^B^
		D ^a/B^	7.3 ^a^	(5.4;9.8) ^a^	1408 ^B^	(1346;1472) ^B^
**3-point**	before	C ^a/A^	8.5 ^a^	(7.0;10.4) ^a^	795 ^A^	(764;827) ^A^
		Z ^a/A^	7.3 ^a^	(5.7;9.5) ^a^	804 ^A^	(768;841) ^B^
		D ^a/A^	9.3 ^a^	(7.3;11.8) ^a^	782 ^A^	(755;811) ^B^
	after	C ^b/AB^	16.5 ^b^	(14.1;19.1) ^b^	1153 ^B^	(1129;1177) ^B^
		Z ^a/A^	8.5 ^a^	(7.1;10.2) ^a,b^	1097 ^B^	(1055;1142) ^A,B^
		D ^a/B^	7.9 ^a^	(6.0;10.4) ^a^	1255 ^B^	(1204:1308) ^B^
**4-point**	before	C ^a/A^	9.1 ^a^	(7.4;11.1) ^a^	591 ^A^	(570;614) ^B^
		Z ^a/B^	8.8 ^a^	(7.0;11.0) ^a^	681 ^A^	(656;708) ^A^
		D ^a/B^	7.1 ^a^	(5.8;8.6) ^a^	679 ^A^	(648;712) ^A^
	after	C ^a/A^	8.4 ^a^	(6.4;11.1) ^a^	923 ^B^	(888;959) ^A^
		Z ^a/A^	5.1 ^a^	(3.9;6.7) ^a^	1002 ^B^	(940;1069) ^A^
		D ^a/A^	5.7 ^a^	(4.2;7.7) ^a^	1023 ^B^	(965;1084) ^A^

^a,b,c^ significant differences for m-values; column ZM indicates significant difference between ZM within TM and SP; column m indicates significant difference between SP within TM and ZM; column 95% CI(m) indicates significant difference between TM within SP and ZM; ^A,B,C^ significant differences for *s*-values; column ZM indicates significant difference between ZM within TM and SP; column s indicates significant difference between SP within TM and ZM; column 95% CI (s) indicates significant difference between TM within SP and ZM.
